# Supplementary material for: HIV-Induced Thymic Insufficiency and Aging-Related Immunosenescence on Immune Reconstitution in ART-Treated Patients
Source: Vaccines (Basel). 2024 Jun 4;12(6):612. doi: 10.3390/vaccines12060612 (PMC11209262; doi:10.3390/vaccines12060612)
Supplement: Supplementary file 1 [file vaccines-12-00612-s001.zip › vaccines-2935700-supplementary.pdf]

Supplementary Table S1. Clinical characteristics of ART-treated HIV-positive patients included in the study.

Variables		INR n=13 (%)	IR n=31 (%)	*P*
Sex	Male	11 (84.6)	22 (71.0)	0.461[a]
	Female	2 (15.4)	9 (29.0)	
Age (years old) at ART start date*		41.8 ± 3.0	33.7 ± 2.1	**0.041**
Body mass (kg)*		74.26 ± 4.4	75.3 ± 2.5	0.892
Time (weeks) to ART starting post-diagnosis**		2.0 (1.0 – 9.5)	8.0 (3.0 – 9.0)	0.176
Detailed ART regimens, NRTI + 3TC + (third option)	ABC+3TC+DTG	1 (7.7)	0 (0.0)	0.571[b]
	ABC+3TC+NVP	0 (0.0)	1 (3.2)	
	TDF+3TC+ATV/r	2 (15.4))	4 (12.9)	
	TDF+3TC+LPV/r	0 (0.0)	1 (3.2)	
	TDF+3TC+DTG	7 (53.8)	14 (45.2)	
	TDF+3TC+EFZ	3 (23.1)	11 (35.5)	
ART regimens, stratified by classes	2 NRTI + INI	8 (61.5)	14 (45.2)	0.564[b]
	2 NRTI + IP/r	2 (15.4)	5 (16.1)	
	2 NRTI + NNRTI	3 (23.1)	12 (38.7)	
ART regimen change[c]		3 (23.1)	7 (22.6)	1.000[a]
Pre-treatment CD4+ T-cell count (cells/µL)*		153.9 ± 38.8	554.5 ± 64.9	**<0.001**
Pre-treatment CD8+ T-cell count (cells/µL)*		718.5 ± 251.0	842.5 ± 201.4	0.734
CD4+ T-cell count after 18 months of ART (cells/µL)*		307.9 ± 37.6	816.6 ± 81.8	**<0.001**
RTE CD4+ T-cells (%)*		19.5 ± 6.3	29.9 ± 11.5	**0.012**
Pre-treatment CD4/CD8 ratio*		0.12 ± 0.05	0.60 ± 0.10	**0.010**
CD4/CD8 ratio after 18 months of ART*		0.48 ± 0.08	1.34 ± 0.13	**<0.001**
Pre-CD4+ (%)*		9.9 ± 2.2	25.0 ± 1.7	**<0.001**
Pre-CD8+ (%)*		65.4 ± 8.4	47.1 ± 4.8	**0.008**
CD4+ (%) after 18 months of ART*		19.5 ± 2.1	38.2 ± 1.6	**<0.001**

CD8+ (%) after 18 months of ART*	46.4 ± 3.4	34.4 ± 1.8	**0.001**
Pre-treatment PVL (log10 RNA copies/mL)*	4.7 ± 0.4	4.1 ± 0.2	0.129

* t-test (Shapiro-Wilk test: >0.05), values displayed as mean ± SE.

**Wilcoxon-Mann-Whitney test (Shapiro-Wilk: <0.05), values displayed as median (IQR).

[a]Fisher's exact test.

[b]Chi-squared test.

[c]Change from a NNTRI-containing cART (EFZ) to a PI/r (ATV/r), INI (DTG), or another NNTRI (NVP) -containing ART regimen.

3TC: lamivudine; ABC: abacavir; ART: antiretroviral therapy; ATV/r: ritonavir-boosted atazanavir; AZT: zidovudine; DTG: dolutegravir; EFZ: efavirenz; INI: integrase inhibitor; INR: immunological non-responders; IR: immunological responders; IQR: interquartile range; LPV/r: ritonavir-boosted lopinavir; NNRTI: non–nucleoside reverse transcriptase inhibitor; NRTI: nucleoside reverse transcriptase inhibitor; NVP: nevirapine; PI/r: ritonavir-boosted protease inhibitor; PVL: plasma viral load; SE: standard error; TDF: tenofovir.
